# AV Nodal Reentrant Tachycardia Causing Inappropriate ICD Shocks In A Patient With Arrhythmogenic RV Dysplasia

**Published:** 2009-01-07

**Authors:** Subramanya Prasad, Jayasree Pillarisetti, Subbareddy Vanga, Dhanunjaya Lakkireddy

**Affiliations:** 1 Florence Medical Group, Florence, KY; 2 Bloch Heart Rhythm Center, Mid America Cardiology @ University of Kansas Hospital, Kansas City, KS USA

**Keywords:** Arrhythmogenic right ventricular dysplasia, Implantable cardioverter defibrillator, Inappropriate shocks

## Abstract

We report a patient with an implantable cardioverter defibrillator (ICD) for arrhythmogenic right ventricular dysplasia (ARVD) who received inappropriate shocks for atrioventricular node reentry tachycardia (AVRNT). Patient had multiple shocks for tachycardia with EGM characteristics of very short VA interval and CL of 300 msec. An electrophysiologic (EP) study reproducibly induced typical AVNRT with similar features. The slow AV nodal pathway ablation resolved the ICD shocks. Despite increasingly sophisticated discrimination algorithms available in modern ICDs, the ability to differentiate SVT from VT can be challenging. Our patient received inappropriate shocks for AVNRT. When device interrogation alone is not conclusive, an EP study may be necessary to determine the appropriate therapeutic course.

## Introduction

Arrhythmogenic right ventricular dysplasia (ARVD) is an inherited degenerative disorder involving progressive replacement of the myocardium by fibro fatty tissue. This is seen predominantly in the right ventricle though left ventricular involvement is not uncommon. The fibrofatty replacement provides the substrate for life threatening arrhythmias such as monomorphic ventricular tachycardia, polymorphic ventricular tachycardia (torsades de pointes), ventricular fibrillation and / or electrical storm (≥ 3 episodes of VT or VF within 24 hours requiring electrical cardioversion or defibrillation   warranting ICD placement [1].  Often supraventricular tachycardias (SVT) coexist in patients with known VT. Atrial fibrillation is the most common SVT known to have been responsible for inappropriate ICD shocks.

Modern implantable cardioverter defibrillators (ICDs) are sophisticated devices with arrhythmia discrimination algorithms that reliably differentiate SVT from VT and provide appropriate therapy. We report a patient with ARVD who had inappropriate shocks for AVNRT.

## Case report

**Figure 1:**
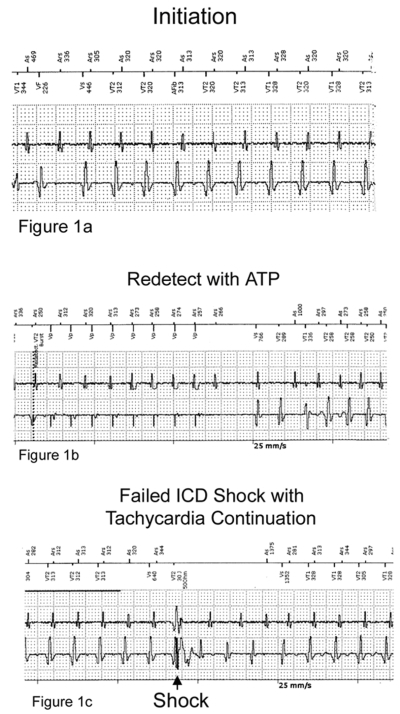
**a - c:** ICD EGMs showing the initiation, antitachycardia pacing and shock therapy followed by the continuation of tachycardia. **1a:** The first beat shows conduction across the fast pathway. The second beat shows conduction across the slow pathway exhibiting dual AV nodal physiology with AV nodal echo and continuation of tachycardia. **1b:** ATP last beat has a retrograde A that goes down the slow pathway anterogradely and and goes back retrogradely through fast pathway and AVNRT continues. **1c:**  ICD shock doesn't terminate the tachycardia.

A 47-year old male presented with 3 ICD shocks for persistent ventricular tachycardia. He had known history of polysubstance abuse and coronary artery disease with angioplasty. Eight months prior to this admission he had presented with symptomatic monomorphic ventricular tachycardia (Cycle length 360 msec). A 2D-echo showed an ejection fraction of 40% followed by a coronary angiogram which showed non-obstructive disease with previously placed patent right coronary stent. Cardiac MRI confirmed a diagnosis of ARVD. He received a Biotronik Lexos DR ICD (model # 347000). Device interrogation during the current admission revealed a wide complex tachycardia (WCT) that received 3 ineffective shocks but resolved spontaneously. The WCT as shown by the Electrograms (EGMs) during ICD interrogation showed a short VA interval, a Cycle length (CL) of 300msec which were completely different from the VT seen during his initial clinical presentation (Figure 1 a-c). ICD shock failed to terminate the WCT which had a spontaneous termination later. Based on these observations, differentiating the arrhythmia (SVT vs VT) was inconclusive thus an electrophysiological study was done.

**Figure 2:**
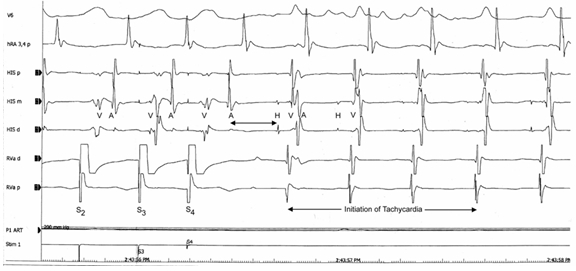
Intracardiac EGMs showing AV nodal jump followed by the initiation of slow-fast AVNRT.

During EP study, no arrhythmia was inducible at baseline. With RV stimulation and Isoproterenol (2mcg) provocation, a typical AVNRT with a pacing cycle length of 500 msecs and 3 extra stimuli of 290-280-270 ms, identical to the arrhythmia seen on ICD interrogation strips was easily and reproducibly induced (Figure 2). Presence of a short VA time (56 milliseconds) excluded an accessory pathway. A constant HA interval with resumption of tachycardia during ventricular pacing maneuvers and no A-A-V response after ventricular overdrive burst pacing excluded atrial tachycardia. The slow AV nodal pathway was ablated with RF energy with 3D-mapping (CARTO, Biosense Webster) for additional guidance. Following ablation, no inducible tachycardia, dual AV nodal physiology or echo beats were seen spontaneously or with Isoproterenol provocation. His pacing and sensing thresholds and lead impedance both in A and V leads seem to be appropriate after ablation. Defibrillation thresholds were unchanged.

## Discussion

Our patient presented with sustained monmorphic VT, was diagnosed as ARVD by cardiac MRI, requiring ICD implantation. Subsequently he had inappropriate shocks due to AVNRT (atrial arrhythmia with a stable atrioventricular relationship). Due to differences in the characteristics of the two arrhythmias an appropriate diagnosis of the arrhythmia responsible for the inappropriate shocks could be established only with programmed electrical stimulation during an EP study.

ARVD has an increased incidence of ventricular arrhythmias and SCD requiring ICD implantation. It has been estimated that about 20 percent of SCD among patients <35 years is due to ARVD [2]. Modern dual chamber ICDs utilize arrhythmia discrimination algorithms that have been shown to be reliable in sensing and terminating atrial and ventricular arrhythmias [3]. Our patient had a Biotronik ICD which utilizes the Smart ®  detection algorithm with 93 percent specificity for atrial arrhythmia discrimination and a100% specificity for ventricular arrhythmia discrimination [4].

In spite of a high specificity, inappropriate shocks have been seen in ARVD patients with ICD especially with atrial arrhythmias. In a study of 653 arrhythmia episodes in patients with dual chamber ICDs, inappropriate detection of atrial arrhythmias (with a stable atrioventricular relationship) was seen in 42% of patients, of which atrial flutter or fibrillation were more often misdiagnosed [5].

## Conclusion

Most modern ICDs have sophisticated discrimination algorithms to differentiate atrial and ventricular arrhythmias. While ventricular arrhythmias are recognized and treated with precision, atrial arrhythmias can be misdiagnosed and receive inappropriate shock therapy. An EP study may thus be necessary in establishing an accurate diagnosis and delivering appropriate therapy.

## Abbreviations

ARVD: Arrhythmogenic Right Ventricular Dysplasia
ICD: Implantable Cardioverter Defibrillator
VPS: Ventricular Programmed Stimulation
AVNRT: Atrioventricular Node Reentry Tachycardia
VT: Ventricular Tachycardia
SVT: Supraventricular Tachycardia

## References

[R1] Credner SC, Klingenheben T, Mauss O (1998). Electrical Storm in patients with transvenous implantable cardioverter-defibrillators. Incidence, management and prognostic implications. J Am Coll Cardiol.

[R2] Calkins H, Marcus F, Braunwald E (2003). Arrhythmogenic right ventricular dysplasia. Harrison's Advances in Cardiology.

[R3] Roguin A, Bomma CS, Nasir K (2004). Implantable cardioverter-defibrillators in patients with arrhythmogenic right ventricular dysplasia/cardiomyopathy. J Am Coll Cardiol.

[R4] Theuns D, Klootwijk AP, Kimman GP (2001). Initial clinical experience with a new arrhythmia detection algorithm in dual chamber implantable cardioverter defibrillators. Europace.

[R5] Theuns DA, Klootwijk AP, Goedhart DM (2004). Prevention of inappropriate therapy in implantable cardioverter-defibrillators: results of a prospective, randomized study of tachyarrhythmia detection algorithms. J Am Coll Cardiol.

